# Development of a predictive risk model for all-cause mortality in patients with diabetes in Hong Kong

**DOI:** 10.1136/bmjdrc-2020-001950

**Published:** 2021-06-11

**Authors:** Sharen Lee, Jiandong Zhou, Keith Sai Kit Leung, William Ka Kei Wu, Wing Tak Wong, Tong Liu, Ian Chi Kei Wong, Kamalan Jeevaratnam, Qingpeng Zhang, Gary Tse

**Affiliations:** 1 Cardiovascular Analytics Group, Laboratory of Cardiovascular Physiology, Hong Kong; 2 School of Data Science, City University of Hong Kong, Kowloon, Hong Kong; 3 Aston Medical School, Aston University, Birmingham, UK; 4 Faculty of Medicine, The Chinese University of Hong Kong, Hong Kong, China; 5 School of Life Sciences, The Chinese University of Hong Kong, Hong Kong, China; 6 Department of Cardiology, The Second Hospital of Tianjin Medical University, Tianjin, China; 7 Li Ka Shing Faculty of Medicine, University of Hong Kong, Hong Kong, China; 8 Faculty of Health and Medical Sciences, University of Surrey, Guildford, Surrey, UK; 9 Kent and Medway Medical School, Canterbury, UK

**Keywords:** epidemiology, risk factors

## Abstract

**Introduction:**

Patients with diabetes mellitus are risk of premature death. In this study, we developed a machine learning-driven predictive risk model for all-cause mortality among patients with type 2 diabetes mellitus using multiparametric approach with data from different domains.

**Research design and methods:**

This study used territory-wide data of patients with type 2 diabetes attending public hospitals or their associated ambulatory/outpatient facilities in Hong Kong between January 1, 2009 and December 31, 2009. The primary outcome is all-cause mortality. The association of risk variables and all-cause mortality was assessed using Cox proportional hazards models. Machine and deep learning approaches were used to improve overall survival prediction and were evaluated with fivefold cross validation method.

**Results:**

A total of 273 678 patients (mean age: 65.4±12.7 years, male: 48.2%, median follow-up: 142 (IQR=106–142) months) were included, with 91 155 deaths occurring on follow-up (33.3%; annualized mortality rate: 3.4%/year; 2.7 million patient-years). Multivariate Cox regression found the following significant predictors of all-cause mortality: age, male gender, baseline comorbidities, anemia, mean values of neutrophil-to-lymphocyte ratio, high-density lipoprotein-cholesterol, total cholesterol, triglyceride, HbA1c and fasting blood glucose (FBG), measures of variability of both HbA1c and FBG. The above parameters were incorporated into a score-based predictive risk model that had a c-statistic of 0.73 (95% CI 0.66 to 0.77), which was improved to 0.86 (0.81 to 0.90) and 0.87 (0.84 to 0.91) using random survival forests and deep survival learning models, respectively.

**Conclusions:**

A multiparametric model incorporating variables from different domains predicted all-cause mortality accurately in type 2 diabetes mellitus. The predictive and modeling capabilities of machine/deep learning survival analysis achieved more accurate predictions.

**Significance of this study**

**What is already known about this subject?**

- Increased variability in metabolic parameters is predictive of higher mortality in type 2 diabetes mellitus.

**What are the new findings?**

- We developed a machine learning-driven predictive risk model for type 2 diabetes mellitus using multiparametric approach with data from different domains.
- Measures of variability of fasting glucose and HbA1c show similar predictive power for all-cause mortality, regardless of whether adjustments were made for initial values or mean values across follow-up.
- A multiparametric predictive risk model incorporating variables from different domains, including baseline demographics, comorbidities and laboratory tests, measures of variability of HbA1c and fasting blood glucose predicted all-cause mortality accurately.
- Machine learning-driven algorithms further improved the accuracy of the predictive models.

**How might these results change the focus of research or clinical practice?**

- A simple, easy-to-use score-based system has been devised to enable rapid risk prediction in the clinical setting.

## Introduction

Type 2 diabetes mellitus is one of the most common metabolic conditions, with an increasing prevalence attributable to aging, sedentary lifestyles, environmental changes and better disease management.^1–3^ Patients with this condition are at an increased risk of premature death and other complications.^4 5^ Existing risk models have been developed, such as QDiabetes for predicting new onset diabetes,^6^ and CORE,^7^ BRAVO^8^ and Michigan^9^ models for predicting disease progression, complications and mortality. These have generated good predictive results in western cohorts but are limited by their direct applicability to Asian populations. For example, Chinese patients have a lower body mass index threshold for diabetes development and have a higher propensity to suffer from chronic kidney disease as a result.^10 11^ While Asian population-specific models are available,^12–15^ these have generally not incorporated temporal measures of variability for longitudinal data or machine learning approaches, both of which can enhance risk prediction.^16 17^ Indeed, with the rapid development of big data analytics, it has become easier to improve discrimination by analyzing complex interactions among variables. Previously, a machine learning-driven approach has demonstrated superior performance for predicting diabetes onset in a Chinese cohort.^18^

In this territory-wide study, with the aid of machine/deep learning approaches, we developed a risk model for mortality prediction using multiparametric data from different domains. These include baseline comorbidities, measures of variability of fasting glucose and HbA1c, inflammatory and nutritional indices and drug prescription details. We tested the hypothesis that machine learning methods (random survival forests, RSF^19^) and deep neural survival learning models (DeepSurv^20^) can significantly improve predictive performance when compared with Cox regression-based models.

## Methods

### Study design and data source

The inclusion criteria were patients who received antidiabetic medications or had International Classification of Disease, Ninth Edition (ICD-9) codes for type 2 diabetes mellitus, and attended any of the 43 public hospitals or their associated ambulatory or outpatient facilities managed by the Hong Kong Hospital Authority between January 1 and December 31, 2009. The Clinical Data Analysis and Reporting System, a healthcare database that integrates patient information to establish comprehensive medical records with accurately linked mortality data were used in this study. This system has been used for epidemiological research by multiple research teams, including our team, in the past,^21–24^ including model development studies.^25 26^

### Data extraction

Baseline patient characteristics include demographic details such as age and sex, prior comorbidities (heart failure (HF), ischemic heart disease (IHD), ischemic stroke, aborted sudden cardiac death (SCD) of all causes including acute myocardial infarction, atrial fibrillation (AF), peripheral vascular disease, intracranial hemorrhage, osteoporosis, dementia, hypertension, chronic obstructive pulmonary disease (COPD), cancer, renal and ophthalmological diabetic complications), antidiabetic and cardiovascular medications. The ICD-9 codes for the aforementioned comorbidities are summarized in online supplemental table 1. The duration of living with type 2 diabetes mellitus from the point of diagnosis until December 31, 2009 was also extracted, and determined by the earliest fulfillment of any of the following criteria in this order: (1) initial documentation of type 2 diabetes mellitus related ICD-9 codes; (2) earliest HbA1c>6.5%; (3) earliest fasting blood glucose (FBG) >7 mmol/L. Time-till all-cause mortality was determined as the number of days from the starting date of patient inclusion, January 1, 2009, until the day of death or the end of the follow-up period, December 31, 2019.

10.1136/bmjdrc-2020-001950.supp1 Supplementary data

The following laboratory data were collected at baseline: neutrophil-lymphocyte ratio (NLR) was derived by dividing the absolute neutrophil by the lymphocyte count, anemia defined as <13 g/dL for men and <12 g/dL for women, biochemical test results including (1) creatinine, sodium, potassium, (2) urea, (3) albumin and total protein, (4) alanine aminotransferase and alkaline phosphatase, (5) FBG and HbA1c; (6) high-density lipoprotein-cholesterol (HDL-C), directly measured low-density lipoprotein-cholesterol (LDL-C), total cholesterol, and triglyceride.

The number of antidiabetic drugs by class were extracted: (1) insulin, (2) biguanide, (3) sulphonylurea, (4) alpha-glucosidase inhibitor, (5) thiazolidinedione, (6) dipeptidyl peptidase-4 inhibitor, (7) glucagon-like peptide receptor-1 agonist, (8) meglitinide. Similarly, the number of antihypertensive medications of the following classes were also extracted: (1) angiotensinogen-converting-enzyme inhibitor/angiotensin receptor blocker, (2) beta-adrenergic receptor blocker, (3) calcium channel blocker, (4) diuretics. Lipid-lowering agents were also extracted.

### Variability calculations

To calculate FBG and HbA1c variability, data points were obtained for the period between January 1, 2004 and December 31, 2008. Only patients with three or more measurements for the specific parameter were included for the variability analysis of the respective parameter. The different measures are detailed below and summarized in online supplemental table 2: (1)SD, (2) absolute variability score defined as 100× no. of measurements>0.5/no. of measurements, (3) percentage variability score defined as 100× no. of measurements>10% of previous measurement/no. of measurements, (4) normalized absolute variability score given by (2)/individual mean, (5) normalized percentage variability score given by (3) /individual mean, (6) SD/individual baseline, (7) coefficient of variation given by SD/individual mean, (8) variability independent of mean given by SD/individual meanˆ(ln(population SD)/ln(population mean)).

### Outcomes and statistical analysis

The primary outcome for the present student is all-cause mortality. Univariate Cox regression was applied to identify significant predictors for all-cause mortality and HR with 95% CI were reported. Variables achieving p<0.10 were included in a diabetes duration-adjusted multivariate model. Statistical significance is defined as p<0.05. FBG and HbA1c variability of the same formula were paired and added to the multivariate model to assess their predictiveness through comparison of HR, thus preventing problems with collinearity.

To generate a predictive score, Cox regression was repeated for the final multivariate model with measures of variability included. HR between 1 and 1.50 was awarded 1 mark in the score. To adjust for the U-shaped relationship against mortality reported for HDL-C, LDL-C, total cholesterol and HbA1c, these parameters were first divided by deciles to serve as cut-offs and undergo univariate Cox regression. Thereafter, the decile with the smallest HR was selected as the reference and compared against the remaining deciles through univariate Cox regression again. The minimum and maximum cut-offs for the deciles that had an insignificant difference with the reference decile were selected as the cut-offs to be used in the score. To demonstrate the U-shaped relationship, the HR of deciles was plotted graphically. Similar methods were employed to illustrate the U-shaped relationship by existing studies.^27–29^ Cut-off values for continuous variables in the score were found through maximizing sensitivity and specificity. Age and diabetes duration were rounded to the nearest whole number, while other parameters were rounded to two decimal points. The predictive value of the score was evaluated through the generation of a receiver operating characteristic (ROC) curve and area under the-curve (AUC) calculated.

To further evaluate the predictive value of the measures of variability, the measures were also divided into quartiles, with the first quartile as a reference, to perform univariate Cox regression and assess the AUC of the quartile cut-offs. The quartile HR of the FBG and HbA1c measures of variability were illustrated graphically. Statistical analyses were performed using RStudio software (V.1.1.456) and Python (V.3.6).

### Development of machine/deep models for survival learning

Machine/deep learning survival analysis models can directly capture the relationships between risk predictors and mortality outcome without prior functional assumptions typically made in Cox analysis models. Here we used an RSF model, a type of machine learning method for survival analysis, relying on the intuition that the best survival learning model, when combined with weak decision tree learning models, can minimize the overall survival prediction errors. The prediction errors are measured by performance evaluators, for example, precision, recall, AUC and C-index. The out-of-bag (OOB) method was adopted whenever a bootstrap sample (bag ones) is down with replacement from the training dataset. The bootstrapping technique is used to grow the tree and results in well-defined subsets. Some of the bootstrap are duplicates and are members of the in-bag subset, and the remaining individuals define the OOB subset for the final tree. Each individual in the OOB subset for a tree is passive. A unique terminal node membership and terminal node statistic were assigned. An OOB ensemble statistic for each individual is formed by combining the terminal node statistics from all trees where an individual is an OOB member. Finally, the class with the maximum frequency in the OOB ensemble statistic serves as the predicted class label for the member. More detailed descriptions of these concepts were described by Breiman *et al*.^30^

The variable’s importance of interest is calculated as the prediction error (squared loss) of the original ensemble event-specific cumulative probability function subtracted from the prediction error of the original ensemble event-specific cumulative probability function (obtained when each OOB instance is just dropped down its in-bag competing risks tree).^31 32^ In this study, RSF was used for mortality prediction and the most important predictors were ranked according to variable importance measure in RSF. Variables that were important predictors of risk outcome have a larger importance value, indicating higher predictive strength, whereas non-predictive variables have zero or negative values.

We further employed a nonlinear deep learning survival method termed Cox proportional hazards DeepSurv approach. This can inherently and adaptively model the high-level interaction patterns among risk predictors and thus can better capture the complex nonlinear relationship between patients’ covariates (eg, clinical features) and mortality outcome directly. Specifically, DeepSurv is a deep feed-forward neural network that can predict the effects of a patient’s baseline covariates on their hazard rate parameterized by the weights of the neural network. The input of DeepSurv is the baseline variables of the patient with diabetes. The hidden layers of DeepSurv consist of a fully connected layer of nodes, followed by a dropout layer.^33^ The output of the DeepSurv is a single node with a linear activation which estimates the log-risk function in the Cox model. In this study, we train DeepSurv by presetting the objective function to be the average negative log form of Cox partial likelihood with L2-regularization,^34^ in order to model for mortality risk prediction of the patients with diabetes. Gradient descent optimization was used to find the weights of DeepSurv. The hyperparameters of DeepSurv including the number of hidden layers, the number of nodes in each layer and dropout probability were determined from a random hyperparameter search approach.^35^

The RSF, DeepSurv and multivariate Cox regression models adopted the same set of predictors. A fivefold cross-validation approach was performed to compare the survival prediction performance of RSF and DeepSurv in terms of precision, recall, AUC and HC-index over the standard Cox model. The R packages, *randomForestSRC* (V.2.9.3), *ggplot2* (V.3.3.2), and python package *DeepSurv* (V.0.1.0) were used to generate the mortality prediction results.

## Results

### Baseline characteristics

The study cohort included 273 876 patients (mean age: 65.4±12.7 years, male: 48.2%, diabetes duration=6.18 ± 4.56 years) with a median follow-up of 142 (IQR (IQR)=106–142) months, which corresponded to a total of 2 660 465 patient-years. The baseline demographics, clinical, laboratory and drug details are shown in tables 1 and 2 for continuous and discrete variables, respectively. The most prevalent comorbidities were hypertension, IHD and HF. The percentage of patients on n=0, 1, 2, 3, and 4 antidiabetic medications were 13.3%, 34.8%, 46.1%, 5.4% and 0.4%, respectively. At baseline, the fasting glucose and HbA1c were 8.02±1.95 mmol/L and 7.75%±2.59%, respectively. The median number for fasting glucose and HbA1c measurements were 7 (IQR=4–11) and 7 (IQR=4–10), respectively. The different measures of variability for fasting glucose or HbA1c are quantified for subsequent use to predict mortality (detailed methodology is shown in online supplemental table 2.

**Table 1:** Baseline characteristics for continuous variables

Characteristics	Mean	SD
Age	65.4	12.7
Follow-up duration (days)	3546	1208
Diabetes duration (years)	6.18	4.56
Liver function test		
Alkaline phosphatase (U/L)	81.1	37.6
Alanine aminotransferase (U/L)	28.8	52.9
Total protein (g/L)	74.5	6.67
Albumin (g/L)	38.9	5.04
Complete blood count		
Lymphocyte count (×10^9^/L)	1.89	1.04
Neutrophil count (×10^9^/L)	5.35	2.69
Neutrophil-lymphocyte ratio	3.72	4.37
Hemoglobin count (×10^9^/L)	12.8	1.86
Lipid profile		
HDL-C (mmol/L)	1.23	0.348
LDL-C (mmol/L)	3.09	0.941
Total cholesterol (mmol/L)	5.12	1.13
Triglyceride (mmol/L)	1.63	1.51
Renal function test		
Creatinine (umol/L)	102	87.2
Potassium (mmol/L)	4.24	0.522
Sodium (mmol/L)	139	3.48
Urea (mmol/L)	6.96	4.11
Glycemic control		
Fasting blood glucose	8.02	1.95
HbA1c	7.75	2.59

HDL-C, high-density lipoprotein-cholesterol; LDL-C, low-density lipoprotein-cholesterol.

**Table 2:** Baseline characteristics for discrete variables

Characteristics	Number	Percentage
Male	132 040	48.2
Baseline anemia	39 799	14.5
Antidiabetic agent		
Biguanide	185 881	67.9
Sulphonylurea	173 525	63.4
Insulin	29 697	10.8
Meglitinide	27	0.01
Dipeptidyl peptidase-4 inhibitor	325	0.12
Thiazolidinedione	3698	1.35
Glucagon-like peptide-1 agonist	17	0.006
Acarbose	3292	1.20
Cardiovascular drugs		
ACEI/ARB	121 786	44.5
Beta-adrenergic receptor blocker	92 309	33.7
Calcium channel blocker	109 225	39.9
Diuretic	52 096	19.0
Lipid-lowering agent	61 401	22.4
Comorbidities		
Diabetic renal complication	3381	1.23
PVD	346	0.13
Diabetic ophthalmological complication	3543	1.29
Ischemic Stroke	8986	3.28
SCD	6420	2.34
AF	7772	2.84
HF	11 189	4.09
Intracranial hemorrhage	3264	1.19
IHD	26 423	9.65
Osteoporosis	137	0.050
Dementia	2842	1.04
Hypertension	64 246	23.5
Chronic obstructive pulmonary disease	818	0.299
Cancer	12 190	4.45

ACEI, angiotensinogen converting enzyme inhibitor; AF, atrial fibrillation; ARB, angiotensin receptor blocker; HF, heart failure; IHD, ischemic heart disease; PVD, peripheral vascular disease; SCD, sudden cardiac death.

### Predictors of all-cause mortality

Over a median follow-up period of 142 (IQR=106–142) months, 91 155 deaths were recorded (33.3%), which corresponded to an annualized mortality rate of 3.43%. The significant univariate predictors for all-cause mortality are presented in table 3. All measures of variability for FBG and HbA1c were significant predictors as well. The graphical comparison of HR from quartile cut-offs of FBG and HbA1c variability predictors are shown in online supplemental figure 1A, B, with the details summarized in online supplemental tables 3 and 4.

**Table 3:** Univariate predictors for all-cause mortality

	HR	95% CI	P value
Age	1.090	(1.089 to 1.091)	<0.0001
Male	1.12	(1.11 to 1.14)	<0.0001
Complete blood count			
Neutrophil-lymphocyte ratio	1.033	(1.032 to 1.034)	<0.0001
Baseline anemia	3.50	(3.45 to 3.55)	<0.0001
Lipid profile			
HDL-C	0.836	(0.815 to 0.857)	<0.0001
LDL-C	0.883	(0.874 to 0.892)	<0.0001
Total cholesterol	0.910	(0.903 to 0.916)	<0.0001
Triglyceride	0.963	(0.957 to 0.970)	<0.0001
Comorbidity			
Renal diabetic complication	3.68	(3.54 to 3.83)	<0.0001
Ophthalmological diabetic complication	2.73	(2.62 to 2.84)	<0.0001
Peripheral vascular disease	4.39	(3.91 to 4.93)	<0.0001
Ischemic stroke	2.85	(2.78 to 2.93)	<0.0001
Sudden cardiac death	2.48	(2.40 to 2.56)	<0.0001
Atrial fibrillation	3.54	(3.45 to 3.64)	<0.0001
Heart failure	4.74	(4.64 to 4.85)	<0.0001
Intracranial hemorrhage	2.70	(2.59 to 2.82)	<0.0001
Ischemic heart disease	2.24	(2.20 to 2.28)	<0.0001
Osteoporosis	2.87	(2.34 to 3.52)	<0.0001
Dementia	5.92	(5.69 to 6.16)	<0.0001
Hypertension	2.55	(2.52 to 2.59)	<0.0001
Chronic obstructive pulmonary disease	4.55	(4.22 to 4.91)	<0.0001
Cancer	2.48	(2.42 to 2.54)	<0.0001
FBG			
Mean	1.00	(0.997 to 1.01)	0.527
Absolute successive variability score	1.008	(1.007 to 1.008)	<0.0001
Percentage successive variability score	1.01	(1.009 to 1.01)	<0.0001
SD	1.15	(1.15 to 1.16)	<0.0001
Normalized absolute successive variability score	1.065	(1.06 to 1.07)	<0.0001
Normalized percentage successive variability score	1.07	(1.067 to 1.074)	<0.0001
SD/initial FBG	1.01	(1.009 to 1.01)	<0.0001
Coefficient of variation	1.019	(1.018 to 1.019)	<0.0001
Variability independent of mean	1.011	(1.01 to 1.011)	<0.0001
HbA1c			
Mean	1.07	(1.06 to 1.07)	<0.0001
Absolute successive variability score	1.01	(1.007 to 1.01)	<0.0001
Percentage successive variability score	1.008	(1.08 to 1.009)	<0.0001
SD	1.19	(1.18 to 1.20)	<0.0001
Normalized absolute successive variability score	1.055	(1.05 to 1.06)	<0.0001
Normalized percentage successive variability score	1.063	(1.06 to 1.07)	<0.0001
SD/initial HbA1c	1.014	(1.01 to 1.014)	<0.0001
Coefficient of variation	1.017	(1.016 to 1.018)	<0.0001
Variability independent of mean	1.011	(1.01 to 1.012)	<0.0001

FBG, fasting blood glucose; HDL-C, high-density lipoprotein-cholesterol; LDL-C, low-density lipoprotein cholesterol.

**Figure 1:**
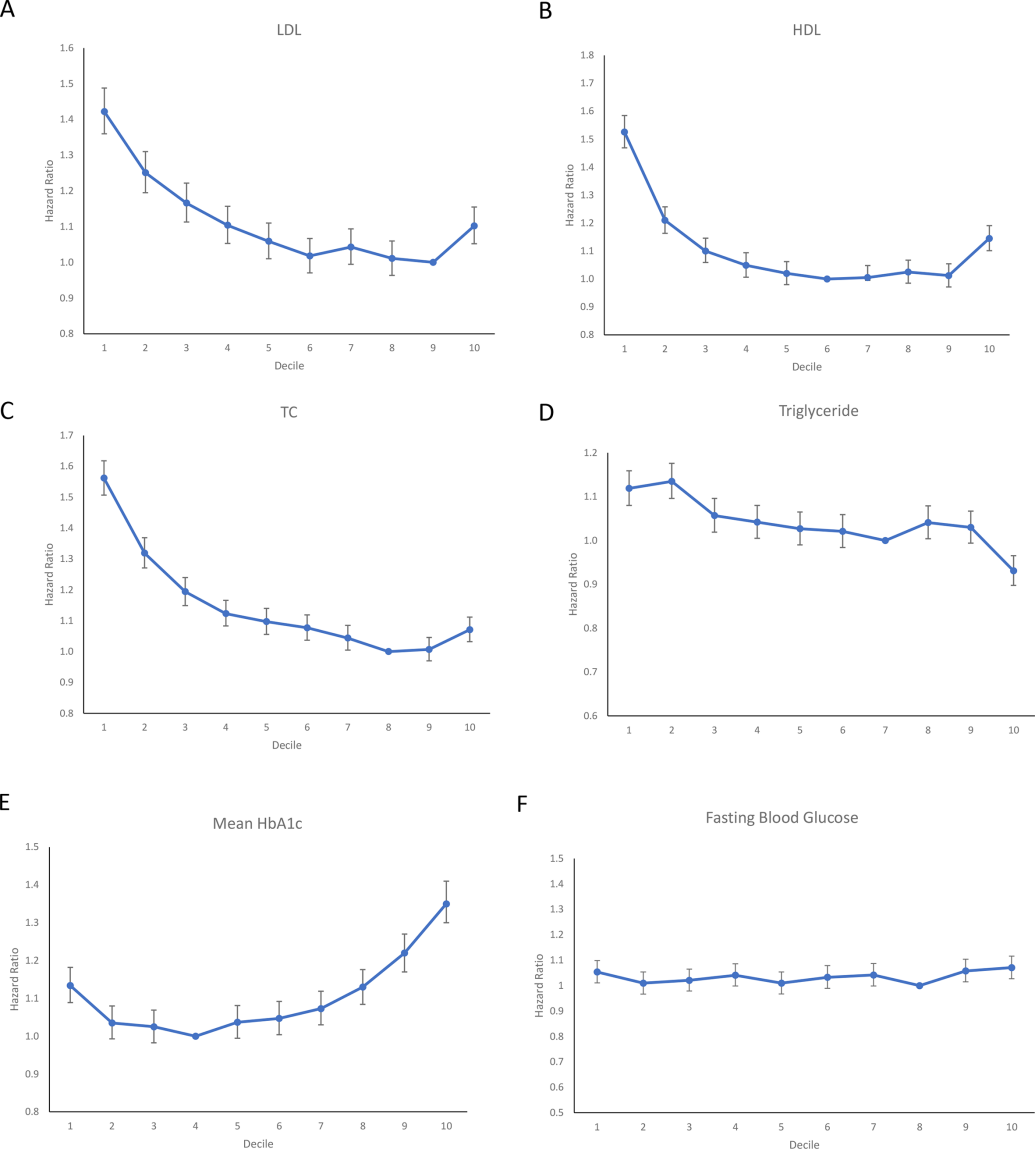
Graphical representation of HRs for all-cause mortality: (A) LDL-C, (B) HDL-C, (C) total cholesterol, (D) triglyceride, (E) mean HbA1c, (F) mean fasting blood glucose. HDL-C, high-density lipoprotein-cholesterol; LDL-C, low-density lipoprotein-cholesterol.

The following parameters remained significant predictors following multivariate adjustment (table 4): (1) age and male gender, baseline comorbidities or complications (hypertension, HF and AF, COPD, cancer, dementia, ischemic stroke, intracranial hemorrhage, aborted SCD, diabetic renal and ophthalmological complications), (2) laboratory tests (anemia, neutrophil-to-lymphocyte ratio (NLR); HDL-C, total cholesterol, triglyceride; mean HbA1c and mean FBG), (3) eight different measures of variability for HbA1c and FBG (table 4). A U-shaped relationship between HDL-C, LDL-C, total cholesterol (figure 1A–C), but not for triglyceride (figure 1D) and all-cause mortality. A U-shaped relationship was also observed for HbA1c but not for FBG (figure 1E, F).

**Table 4:** Multivariate Cox regression for all-cause mortality

	HR	95% CI	P value
Age	1.06	(1.06 to 1.06)	<0.0001
Male	1.35	(1.31 to 1.40)	<0.0001
Complete blood count			
Neutrophil-lymphocyte ratio	1.02	(1.02 to 1.03)	<0.0001
Baseline anemia	1.94	(1.87 to 2.01)	<0.0001
Lipid profile			
High-density lipoprotein cholesterol	0.891	(0.849 to 0.935)	<0.0001
Low-density lipoprotein cholesterol	1.01	(0.986 to 1.04)	0.348
Total cholesterol	1.04	(1.01 to 1.06)	0.001
Triglyceride	1.02	(1.01 to 1.03)	0.001
Comorbidity			
Renal diabetic complication	1.28	(1.20 to 1.36)	<0.0001
Ophthalmological diabetic complication	1.18	(1.11 to 1.26)	<0.0001
Peripheral vascular disease	1.16	(0.984 to 1.37)	0.078
Ischemic stroke	1.25	(1.18 to 1.32)	<0.0001
Sudden cardiac death	1.17	(1.09 to 1.25)	<0.0001
Atrial fibrillation	1.30	(1.23 to 1.37)	<0.0001
Heart failure	1.62	(1.54 to 1.69)	<0.0001
Intracranial hemorrhage	1.28	(1.16 to 1.41)	<0.0001
Ischemic heart disease	1.01	(0.971 to 1.05)	0.574
Osteoporosis	1.03	(0.769 to 1.38)	0.842
Dementia	1.81	(1.64 to 2.00)	<0.0001
Hypertension	1.30	(1.26 to 1.35)	<0.0001
Chronic obstructive pulmonary disease	1.43	(1.20 to 1.70)	<0.0001
Cancer	1.41	(1.33 to 1.49)	<0.0001
Mean FBG	1.01	(1.00 to 1.02)	0.011
Mean HbA1c	1.06	(1.04 to 1.08)	<0.0001
FBG			
Absolute successive variability score	1.00	(1.00 to 1.00)	0.033
Percentage successive variability score	1.00	(1.00 to 1.00)	<0.0001
SD	1.08	(1.07 to 1.10)	<0.0001
Normalized absolute successive variability score	1.02	(1.01 to 1.02)	<0.0001
Normalized percentage successive variability score	1.03	(1.02 to 1.03)	<0.0001
SD/initial	1.00	(1.00 to 1.00)	<0.0001
Coefficient of variation	1.01	(1.01 to 1.01)	<0.0001
Variability independent of mean	1.01	(1.01 to 1.01)	<0.0001
HbA1c			
Absolute successive variability score	1.00	(1.00 to 1.00)	<0.0001
Percentage successive variability score	1.00	(1.00 to 1.00)	<0.0001
SD	1.11	(1.07 to 1.14)	<0.0001
Normalized absolute successive variability score	1.02	(1.01 to 1.03)	<0.0001
Normalized percentage successive variability score	1.03	(1.02 to 1.04)	<0.0001
SD/initial	1.01	(1.01 to 1.01)	<0.0001
Coefficient of variation	1.01	(1.00 to 1.01)	<0.0001
Variability independent of mean	1.00	(1.00 to 1.01)	<0.0001

FBG, fasting blood glucose.

### Development of a score-based predictive risk model based on Cox regression

A score-based predictive risk model for all-cause mortality was developed by incorporating significant predictors from multivariate analysis. One point was allocated for each significant predictor where the HR was less than 1.5, and 2 points for HRs between 1.5 and 2.5. Out of the eight measures of variability for HbA1c and FBG, SD had the highest HR and greatest statistical significance when adjusted to the multivariate model (FBG: HR=1.08, 95% CI 1.07 to 1.10, p<0.0001; HbA1c: HR=1.11, 95% CI 1.07 to 1.14, p<0.0001). It was therefore selected to be included in the mortality score. Altogether, the predictive risk model had a total score out of 25 (table 5). ROC analysis was performed, demonstrating the AUC of 0.729 (95% CI 0.727 to 0.731; online supplemental figure 2). Kaplan-Meier curve and the Kaplan-Meier curve stratified by male gender are shown in online supplemental figure 3 for patients with diabetes. The survival curve generated by the multivariate Cox regression model is shown in online supplemental figure 4.

**Table 5:** A score-based predictive risk model for all-cause mortality in type 2 diabetes mellitus

	Criteria	Score
Age	>70	1
Male	Male	1
Complete blood count		
Neutrophil-lymphocyte ratio	>2.85	1
Baseline anemia	Present	2
Lipid profile		
High-density lipoprotein-cholesterol (mmol/L)	<1.10 or>1.67	1
Total cholesterol (mmol/L)	<5.60 or>6.50	1
Total triglyceride (mmol/L)	>1.24	1
Comorbidity		
Renal diabetic complication	Present	1
Ophthalmological diabetic complication	Present	1
Peripheral vascular disease	Present	1
Ischemic stroke	Present	1
Sudden cardiac death	Present	1
Atrial fibrillation	Present	1
Heart failure	Present	1
Intracranial hemorrhage	Present	1
Dementia	Present	2
Hypertension	Present	1
Chronic obstructive pulmonary disease	Present	1
Cancer	Present	1
Fasting blood glucose and HbA1C: baseline mean and measures of variability		
Mean HbA1c (%)	<6.34 or>7.52	1
Mean FBG (mmol/L)	>6.12	1
SD: FBG	>1.63	1
SD: HbA1c	>0.79	1

Cut-off for age is rounded to the nearest whole number.

FBG, fasting blood glucose.

### Results of machine/deep learning approaches for risk modeling

A RSF model was further performed to predict mortality outcome. The optimal tree number of the RSF model selected as 400 using a fivefold cross-validation approach to minimize the overall squared error rate in the testing set is shown in online supplemental figure 5. In addition, as shown in online supplemental figure 6 about the detailed main results of using the RSF model to predict the mortality outcome of patients with diabetes, the overall ensemble survivals (top left panel) are indicated by the red line and the Nelson-Aalen estimator is given by the green line. Brier score (0=perfect, 0.25>worse than guessing) stratified by ensemble mortality based on the inverse probability of censoring weight method is shown in the top right panel. We stratify the cohort into four groups of 0–25, 25–50, 50–75 and 75–100 percentile mortality (the overall, non-stratified, Brier score is shown by the red line). Continuous rank probability score given by the integrated Brier score divided by time is shown in the bottom left panel, while the illustration of mortality of each patient with diabetes versus observed time of mortality event was shown in the bottom right panel. The mortality events are shown as blue points, and we indicated censored observations using red points. Predicted OOB survivals and the cumulative hazard using the RSF model are shown in online supplemental figure 7. The predicted survival curves of patients with diabetes via the RSF model are shown in online supplemental figure 8 where blue curves correspond to censored observations while red curves represent the observations experiencing mortality events. The 10 most important predictors ranked by the RSF model are shown in online supplemental table 5.

Finally, we compared the survival analysis performance of the RSF model and DeepSurv as typical machine learning and deep learning approaches, respectively, over multivariate Cox model to predict the mortality outcome of the patients with diabetes using the fivefold cross-validation method. Sobol solver^36^ was used to sample each hyperparameter of DeepSurv from a predefined range and k-means cross-validation (k=3) was used to evaluate the performance of the parameter configuration settings. For k-means cross-validation, the dataset was split into k subsets with one subset used as the test set and the remaining as the training set to measure the prediction error. The role of the test and training set was switched until all subsets have been used as the test set, and a mean prediction error would be derived.^37^ Using the configuration with the largest validation C-index on the testing set to avoid models that overfit, we selected the best hyperparameters of the DeepSurv network which included: number of dense layers=4, learning rate=0.0003, ℓ2 regularization coefficient=3.25, dropout rate=0.36, exponential learning rate decay constant=0.0005 and momentum=0.86. In all instances, the ReLU activation function was applied.^38^

The comparative performance results of the different models are shown in table 6. Both RSF and DeepSurv models significantly outperform the multivariate Cox model (precision: 0.85 (95% CI 0.81 to 0.89), recall: 0.86 (0.82 to 0.89), AUC: 0.85 (0.82 to 0.91), C-index: 0.86 (0.81 to 0.90) for DeepSurv model, while precision: 0.85, recall: 0.87, AUC: 0.86, C index: 0.87 for RSF model) based on the same validation inputs of the risk predictors (P for trend <0.001). In addition, the Cox score (precision: 0.88 (0.83 to 0.92), recall: 0.87 (0.84 to 0.91), AUC: 0.86 (0.82 to 0.89), C-index: 0.87 (0.84 to 0.91)) demonstrated better performance than multivariate Cox model (precision: 0.75 (0.72 to 0.79), recall: 0.73 (0.67 to 0.77), AUC: 0.73 (0.68 to 0.76), C-index: 0.73 (0.66 to 0.77)). The advantages of machine/deep learning approaches over the Cox model arise from the fact of their strength to describe survival data with both linear and nonlinear effects from covariates. However, it should be noted that in comparison to DeepSurv, RSF allows influential predictors to be identified more easily by generating an ‘importance ranking’ of the variables with standard bootstrap theory. This enables the investigation of the predictive strength of associated risk predictors for clinicians to estimate the mortality probability just referring to the most important variables.

**Table 6:** Survival prediction performance comparison between Cox, RSF and DeepSurv model with fivefold cross-validation approach

Model	Precision (95% CI)	Recall (95% CI)	AUC (95% CI)	C-index (95% CI)
Multivariate Cox	0.75 (0.72 to 0.79)	0.73 (0.67 to 0.77)	0.73 (0.68 to 0.76)	0.73 (0.66 to 0.77)
Cox score	0.78 (0.74 to 0.82)	0.79 (0.74 to 0.83)	0.77 (0.73 to 0.81)	0.76 (0.72 to 0.82)
DeepSurv	0.85 (0.81 to 0.89)	0.86 (0.82 to 0.89)	0.85 (0.82 to 0.91)	0.86 (0.81 to 0.90)
RSF	0.88 (0.83 to 0.92)	0.87 (0.84 to 0.91)	0.86 (0.82 to 0.89)	0.87 (0.84 to 0.91)

AUC, area under the curve; RSF, random survival forests.

## Discussion

In this study, we developed a machine learning-driven predictive risk model for type 2 diabetes mellitus using a multiparametric approach with data from different domains. Our novel findings are that (1) measures of variability of fasting glucose and HbA1c show similar predictive power for all-cause mortality, regardless of whether adjustments were made for initial values or mean values across follow-up; (2) a multiparametric predictive risk model incorporating variables from different domains, including baseline demographics, comorbidities and laboratory tests, measures of variability of HbA1c and FBG predicted all-cause mortality accurately and (3) machine learning-driven algorithms further improved the accuracy of the predictive models.

Numerous factors have been associated with premature mortality in patients with type 2 diabetes mellitus. Prior epidemiological studies have identified key risk factors including age, comorbidities, healthcare utilization patterns and laboratory findings.^39 40^ In our study, we also identified similar predictors that included advanced age, male gender, high neutrophil and low lymphocyte count, increased levels of urea, creatinine and potassium, as well as reduced levels of HDL-C, LDL-C, triglycerides, total cholesterol and sodium. Moreover, U-shaped relationships between LDL-C, HDL-C and total cholesterol were found in our cohort. These findings are in keeping with U-shaped relationships between cholesterol and all-cause mortality^41^ and for LDL-C^42^ in the general Korean populations. Similar relationships were found for HDL-C, where extremely high LDL-C levels were paradoxically associated with higher mortality.^43^ The association between all-cause mortality and elevated creatinine, urea and potassium, which are classic features of renal failure, is supported by evidence suggesting that the Asian population has a higher risk of developing diabetic nephropathy compared with Caucasians.^11^ It is widely accepted that current predictive models that have largely been developed using Western cohorts only provide moderate levels of accuracy and at times do not lend themselves relevant to disease management protocols that vary by country. Development of country/territory-specific risk prediction models allows for local population-based confounders and clinician management approaches to be incorporated into these models thus providing a more accurate risk prediction for the local population.

Diabetes mellitus is characterized by the presence of systemic chronic inflammation, which is accompanied by increased oxidative stress. To quantify the degree of inflammation, the NLR has been used as a surrogate measure, as it reflects the balance between proinflammatory and anti-inflammatory pathway activation. In our cohort, we found that raised NLR was associated with all-cause mortality risk. We extend previous findings of our group and other groups that increased NLR has been associated with insulin resistance in patients with newly diagnosed type 2 diabetes,^44^ the progression of diabetic nephropathy^45^ and complications in diabetes.^46^ Consequently, the increased oxidative stress environment in diabetes can induce adverse remodeling of the heart, which in turn increases the risk of HF, arrhythmias and cardiovascular mortality.^47 48^

Glycemic variability refers to the fluctuations in glucose levels and can be measured as a daily variation or variation between different clinical visits.^49 50^ Similarly, variability in HbA1c levels has been quantified. Both measures have been associated with a higher risk of complications and mortality in patients with diabetes mellitus in both randomized controlled trials and real-world settings.^51–55^ There are several methods that can be used to calculate variability, such as SD, CV and score based on the frequency exceeding a fixed percentage change in the absolute values. Prior studies have demonstrated the importance of such measures of variability in the prediction of adverse outcomes,^16 17^ but a systematic and direct comparison of different methodologies has not been made with regard to their predictive performance. In our study, eight different measures of variability for HbA1c and FBG were compared, all of which showed significant predictive values. Our findings illustrate that temporal variability in these laboratory tests is important, regardless of the methodology employed for its calculation. In our study, we also found that mean FBG did not predict mortality. Instead, all of the different measures of its variability were all predictive, suggesting that it is intermittent poor glucose control rather than chronic hypoglycemia that are more closely associated with all-cause mortality.

Standard survival model such as Cox proportional hazards model is a semiparametric analysis model to calculate the effects of observed patient’s covariates on the mortality risk outcome. The Cox model assumes the effect of each covariate is proportional. However, in many practical applications, the assumption is not true and risks losing decision information among the observed patient’s covariates. Furthermore, it cannot account for the presence of U-shaped relationships as only a single HR is derived for each covariate. Therefore, numerous nonlinear survival models were developed to better fit survival data with nonlinear log-risk functions (eg, time-encoded methods^56^) or learning the nonlinear relationship directly using machine learning and deep learning techniques (eg, feed-forward neural network risk-predicting methods^57^). RSF model^19^ that is constructed by an ensemble of binary decision trees has been identified as an alternative approach to Cox proportional hazard model in analyzing time-to-event survival data when the linear proportional hazard assumption is violated. DeepSurv^58^ whose multilayer perceptron architecture is deeper than Faraggi-Simon’s feed-forward model and minimizes the negative log Cox partial likelihood with a risk not necessarily linear, is capable to efficiently learn complex non-linear relationships between patient’s covariates and mortality outcome. For model selection among traditional Cox model, the Cox-based score model, RSF, and DeepSurv in risk prediction tasks, there exists a tradeoff: (1) traditional Cox models (as well as Cox based score models) provide good model interpretation ability but less accurate predictions since they sacrificed the consideration of nonlinear inter-dependent patterns among the variables; (2) machine learning or deep learning-based models significantly improves prediction performance especially when the size of instance cohort is rather large (n>1000) but some (eg, DeepSurv) may not provide good interpretations about the resulting predictions. Prediction accuracy and model interpretability are the two most important considerations for risk prediction model selection for clinical use. This study demonstrates the superiority of adopting RSF model for the risk prediction due to both its highest prediction accuracy and good model interpretability.

The findings of this study illustrate that machine/deep survival learning models can better capture the highly complex and nonlinear relationships between prognostic variables and an individual patient’s risk of mortality without prior variable selection or domain knowledge, compared with the traditional Cox analysis model. Application of machine/deep learning to survival analysis performs much better than the standard Cox model in predicting mortality risk of patients with diabetes mellitus. Additionally, machine/deep survival learning models will enable clinicians to provide personalized survival estimations based on the computed probability of mortality risk. In practice, medical researchers can use machine/deep survival learning models to improve overall survival prediction performance based on prognostic characteristics of the patients with diabetes mellitus and subsequently inform early efficient treatment options and even reduce mortality risk.

### Strengths and limitations

The following strengths of our study should be noted. First, this was a territory-wide study with large patient numbers with complete and long follow-up of mortality over 10 years, owing to the linkage of the electronic health records to the death registry. Second, the availability of different data types including prior comorbidities, laboratory test results that included longitudinal data and drug details meant that we were able to build a comprehensive risk model for accurate prediction. Third, the application of the latest machine learning techniques was able to further improve the risk predictions of the models.

However, there are some limitations that should be noted. First, this was a retrospective study and therefore carries the potential bias, such as information bias, that is found in all studies of this type. Second, as with all studies using administrative databases, undercoding is a possibility. This was nevertheless mitigated by our definition of diabetes to include patients with the appropriate ICD coding and those who were on any diabetic medication or met the criteria of diabetes by either HbA1c or fasting glucose results. Patients with type 1 diabetes mellitus were not included given a different disease course and pathogenesis. Further research is needed to explore the potential for the present findings to be extrapolated onto patients with type 1 diabetes mellitus. Third, although the deep neural network survival learning approach demonstrates significant potential in providing much more accurate predictions, the model’s weak interpretability becomes the main obstacle for its real application in clinical practices. Investigations of developing interpretable deep survival learning models that provide highly accurate predictions with supportive explanations for patients with diabetes mellitus become our next research concentration.

## Conclusion

A multiparametric model incorporating variables from different domains predicted all-cause mortality accurately in type 2 diabetes mellitus and a machine/deep learning-driven approach provided further improvements for risk prediction.

## Data Availability

Data are available in a public, open access repository. Data are available on reasonable request. An anonymized version of the dataset has been deposited on Zenodo (https://zenodo.org/record/4383385), in fully compliance with University Regulations and Policy on Dataset Deposit and Sharing. For additional information: https://libguides.lib.cuhk.edu.hk/RDM/dataset_deposit.

## References

[1] Ali MK, Seiglie JA, Narayan KMV. Progress in diabetes prevention or epidemiology-or both, or neither? Lancet Diabetes Endocrinol 2021;9:190–1. 10.1016/S2213-8587(20)30433-2 33636103 PMC8010713

[2] Magliano DJ, Chen L, Islam RM, et al. Trends in the incidence of diagnosed diabetes: a multicountry analysis of aggregate data from 22 million diagnoses in high-income and middle-income settings. Lancet Diabetes Endocrinol 2021;9:203–11. 10.1016/S2213-8587(20)30402-2 33636102 PMC10984526

[3] Yu M, Zhan X, Yang Z, et al. Measuring the global, regional, and national burden of type 2 diabetes and the attributable risk factors in all 194 countries. J Diabetes 2021. 10.1111/1753-0407.13159. [Epub ahead of print: 24 Jan 2021]. 33486878

[4] Xiong Z, Liu T, Tse G, et al. A machine learning aided systematic review and meta-analysis of the relative risk of atrial fibrillation in patients with diabetes mellitus. Front Physiol 2018;9:835. 10.3389/fphys.2018.00835 30018571 PMC6037848

[5] Fung ACH, Tse G, Cheng HL, et al. Depressive symptoms, co-morbidities, and glycemic control in Hong Kong Chinese elderly patients with type 2 diabetes mellitus. Front Endocrinol 2018;9:261. 10.3389/fendo.2018.00261 PMC5986894 29896155

[6] Hippisley-Cox J, Coupland C. Development and validation of QDiabetes-2018 risk prediction algorithm to estimate future risk of type 2 diabetes: cohort study. BMJ 2017;359:j5019. 10.1136/bmj.j5019 29158232 PMC5694979

[7] Palmer AJ, Roze S, Valentine WJ, et al. The core diabetes model: projecting long-term clinical outcomes, costs and cost-effectiveness of interventions in diabetes mellitus (types 1 and 2) to support clinical and reimbursement decision-making. Curr Med Res Opin 2004;20 Suppl 1:S5–26. 10.1185/030079904X1980 15324513

[8] Shao H, Fonseca V, Stoecker C, et al. Novel risk engine for diabetes progression and mortality in USA: building, relating, assessing, and validating outcomes (BRAVO). Pharmacoeconomics 2018;36:1125–34. 10.1007/s40273-018-0662-1 29725871 PMC9115843

[9] Zhou H, Isaman DJM, Messinger S, et al. A computer simulation model of diabetes progression, quality of life, and cost. Diabetes Care 2005;28:2856–63. 10.2337/diacare.28.12.2856 16306545

[10] Chan JC, Cheung CK, Cheung MY, et al. Abnormal albuminuria as a predictor of mortality and renal impairment in Chinese patients with NIDDM. Diabetes Care 1995;18:1013–6. 10.2337/diacare.18.7.1013 7555533

[11] Ma RCW, Chan JCN. Type 2 diabetes in East Asians: similarities and differences with populations in Europe and the United States. Ann N Y Acad Sci 2013;1281:64–91. 10.1111/nyas.12098 23551121 PMC3708105

[12] Ha KH, Lee YH, Song SO, et al. Development and validation of the Korean diabetes risk score: a 10-year national cohort study. Diabetes Metab J 2018;42:402–14. 10.4093/dmj.2018.0014 30113144 PMC6202558

[13] Wang A, Chen G, Su Z, et al. Risk scores for predicting incidence of type 2 diabetes in the Chinese population: the Kailuan prospective study. Sci Rep 2016;6:26548. 10.1038/srep26548 27221651 PMC4879553

[14] Luo S, Han L, Zeng P, et al. A risk assessment model for type 2 diabetes in Chinese. PLoS One 2014;9:e104046. 10.1371/journal.pone.0104046 25101994 PMC4125170

[15] Quan J, Pang D, Li TK, et al. Risk prediction scores for mortality, cerebrovascular, and heart disease among Chinese people with type 2 diabetes. J Clin Endocrinol Metab 2019;104:5823–30. 10.1210/jc.2019-00731 31287503

[16] Scott ES, Januszewski AS, O'Connell R, et al. Long-Term glycemic variability and vascular complications in type 2 diabetes: post hoc analysis of the field study. J Clin Endocrinol Metab 2020;105 10.1210/clinem/dgaa361. [Epub ahead of print: 01 Oct 2020]. 32766757

[17] Wan EYF, Yu EYT, Chin WY, et al. Age-Specific associations of glycated haemoglobin variability with cardiovascular disease and mortality in patients with type 2 diabetes mellitus: a 10- year cohort study. Diabetes Obes Metab 2020;22:1316–27. 10.1111/dom.14034 32196917

[18] Zhang L, Wang Y, Niu M, et al. Machine learning for characterizing risk of type 2 diabetes mellitus in a rural Chinese population: the Henan rural cohort study. Sci Rep 2020;10:4406. 10.1038/s41598-020-61123-x 32157171 PMC7064542

[19] Ishwaran H, Kogalur UB, Blackstone EH, et al. Random survival forests. Ann Appl Stat 2008;2:841–60. 10.1214/08-AOAS169

[20] Katzman JL, Shaham U, Cloninger A, et al. DeepSurv: personalized treatment recommender system using a COX proportional hazards deep neural network. BMC Med Res Methodol 2018;18:24. 10.1186/s12874-018-0482-1 29482517 PMC5828433

[21] Lau WCY, Chan EW, Cheung C-L, et al. Association between dabigatran vs warfarin and risk of osteoporotic fractures among patients with nonvalvular atrial fibrillation. JAMA 2017;317:1151–8. 10.1001/jama.2017.1363 28324091

[22] Ju C, RWC L, Li KHC HJKF. Comparative cardiovascular risk in users versus non-users of xanthine oxidase inhibitors and febuxostat versus allopurinol users. Rheumatology 2019. 10.1093/rheumatology/kez576 31873735

[23] Zhou J, Wang X, Lee S, et al. Proton pump inhibitor or famotidine use and severe COVID-19 disease: a propensity score-matched territory-wide study. Gut 2020:gutjnl-2020-323668. 10.1136/gutjnl-2020-323668 33277346

[24] Zhou J, Lee S, Guo CL, et al. Anticoagulant or antiplatelet use and severe COVID-19 disease: a propensity score-matched territory-wide study. Pharmacol Res 2021;165:105473. 10.1016/j.phrs.2021.105473 33524539 PMC7846462

[25] Li CK, Xu Z, Ho J, et al. Association of NPAC score with survival after acute myocardial infarction. Atherosclerosis 2020;301:30–6. 10.1016/j.atherosclerosis.2020.03.004 32304975

[26] Zhou J, Lee S, Wang X, et al. Development of a multivariable prediction model for severe COVID-19 disease: a population-based study from Hong Kong. NPJ Digit Med 2021;4:66. 10.1038/s41746-021-00433-4 33833388 PMC8032826

[27] Anyanwagu U, Mamza J, Donnelly R, et al. Relationship between HbA1c and all-cause mortality in older patients with insulin-treated type 2 diabetes: results of a large UK cohort study. Age Ageing 2019;48:235–40. 10.1093/ageing/afy178 30615050

[28] Currie CJ, Peters JR, Tynan A, et al. Survival as a function of HbA(1c) in people with type 2 diabetes: a retrospective cohort study. Lancet 2010;375:481–9. 10.1016/S0140-6736(09)61969-3 20110121

[29] Nichols GA, Joshua-Gotlib S, Parasuraman S. Glycemic control and risk of cardiovascular disease hospitalization and all-cause mortality. J Am Coll Cardiol 2013;62:121–7. 10.1016/j.jacc.2013.04.031 23665365

[30] Breiman L. Bagging predictors. Mach Learn 1996;24:123–40. 10.1007/BF00058655

[31] Ishwaran H. Variable importance in binary regression trees and forests. Electron J Stat 2007;1:519–37. 10.1214/07-EJS039

[32] Breiman L. Random forests. Mach Learn 2001;45:5–32. 10.1023/A:1010933404324

[33] Srivastava N, Hinton G, Krizhevsky A, et al. Dropout: a simple way to prevent neural networks from overfitting. J Mach Learn Res 2014;15:1929–58.

[34] AY N. Feature selection, <i>L</i><sub>1</sub> vs. <i>L</i><sub>2</sub> regularization, and rotational invariance. Proceedings of the twenty-first international conference on Machine learning. Banff, Alberta, Canada: Association for Computing Machinery, 2004.

[35] Bergstra J, Bengio Y. Random search for Hyper-Parameter optimization. J Mach Learn Res 2012;13:281–305.

[36] Fox BL. Algorithm 647: implementation and relative efficiency of Quasirandom sequence generators. ACM Trans Math Softw 1986;12:362–76. 10.1145/22721.356187

[37] Kawamoto T, Kabashima Y. Cross-Validation estimate of the number of clusters in a network. Sci Rep 2017;7:3327. 10.1038/s41598-017-03623-x 28607441 PMC5468368

[38] Schmidt-Hieber J. Nonparametric regression using deep neural networks with ReLU activation function. Annals of Statistics 2017;48.

[39] Griffith KN, Prentice JC, Mohr DC, et al. Predicting 5- and 10-year mortality risk in older adults with diabetes. Diabetes Care 2020;43:1724–31. 10.2337/dc19-1870 32669409 PMC7372062

[40] Lowe G, Woodward M, Hillis G, et al. Circulating inflammatory markers and the risk of vascular complications and mortality in people with type 2 diabetes and cardiovascular disease or risk factors: the advance study. Diabetes 2014;63:1115–23. 10.2337/db12-1625 24222348

[41] Yi S-W, Yi J-J, Ohrr H. Total cholesterol and all-cause mortality by sex and age: a prospective cohort study among 12.8 million adults. Sci Rep 2019;9:1596. 10.1038/s41598-018-38461-y 30733566 PMC6367420

[42] Sung K-C, Huh JH, Ryu S, et al. Low levels of low-density lipoprotein cholesterol and mortality outcomes in Non-Statin users. J Clin Med 2019;8. 10.3390/jcm8101571. [Epub ahead of print: 01 Oct 2019]. PMC6832139 31581520

[43] Madsen CM, Varbo A, Nordestgaard BG. Extreme high high-density lipoprotein cholesterol is paradoxically associated with high mortality in men and women: two prospective cohort studies. Eur Heart J 2017;38:2478–86. 10.1093/eurheartj/ehx163 28419274

[44] Lou M, Luo P, Tang R, et al. Relationship between neutrophil-lymphocyte ratio and insulin resistance in newly diagnosed type 2 diabetes mellitus patients. BMC Endocr Disord 2015;15:9. 10.1186/s12902-015-0002-9 25887236 PMC4357061

[45] Azab B, Daoud J, Naeem FB, et al. Neutrophil-to-lymphocyte ratio as a predictor of worsening renal function in diabetic patients (3-year follow-up study). Ren Fail 2012;34:571–6. 10.3109/0886022X.2012.668741 22452450

[46] Wan H, Wang Y, Fang S, et al. Associations between the neutrophil-to-lymphocyte ratio and diabetic complications in adults with diabetes: a cross-sectional study. J Diabetes Res 2020;2020:6219545 10.1155/2020/6219545 32405503 PMC7206875

[47] Tse G, Yan BP, Chan YWF, et al. Reactive oxygen species, endoplasmic reticulum stress and mitochondrial dysfunction: the link with cardiac arrhythmogenesis. Front Physiol 2016;7:313. 10.3389/fphys.2016.00313 27536244 PMC4971160

[48] Tse G, Lai ETH, Tse V, et al. Molecular and electrophysiological mechanisms underlying cardiac arrhythmogenesis in diabetes mellitus. J Diabetes Res 2016;2016:2848759 10.1155/2016/2848759 27642609 PMC5011530

[49] Gorst C, Kwok CS, Aslam S, et al. Long-Term glycemic variability and risk of adverse outcomes: a systematic review and meta-analysis. Diabetes Care 2015;38:2354–69. 10.2337/dc15-1188 26604281

[50] Roever L, Tse G, Biondi-Zoccai G. Variability of metabolic parameters and risk of heart failure: can it be a marker of incident heart failure? Int J Cardiol 2019;293:183–4. 10.1016/j.ijcard.2019.07.007 31296395

[51] Orozco-Beltrán D, Navarro-Pérez J, Cebrián-Cuenca AM, et al. The influence of hemoglobin A1c levels on cardiovascular events and all-cause mortality in people with diabetes over 70 years of age. A prospective study. Prim Care Diabetes 2020;14:678–84. 10.1016/j.pcd.2020.06.003 32605878

[52] Sheng C-S, Tian J, Miao Y, et al. Prognostic Significance of Long-term HbA_1c_ Variability for All-Cause Mortality in the ACCORD Trial. Diabetes Care 2020;43:1185–90. 10.2337/dc19-2589 32229597

[53] Li S, Nemeth I, Donnelly L, et al. Visit-to-Visit HbA_1c_ Variability Is Associated With Cardiovascular Disease and Microvascular Complications in Patients With Newly Diagnosed Type 2 Diabetes. Diabetes Care 2020;43:426–32. 10.2337/dc19-0823 31727686

[54] Lee S, Liu T, Zhou J, et al. Predictions of diabetes complications and mortality using HbA1c variability: a 10-year observational cohort study. Acta Diabetol 2021;58:171–80. 10.1007/s00592-020-01605-6 32939583

[55] Lee S, Zhou J, Wong WT, et al. Glycemic and lipid variability for predicting complications and mortality in diabetes mellitus using machine learning. BMC Endocr Disord 2021;21:94. 10.1186/s12902-021-00751-4 33947391 PMC8097996

[56] Jerez JM, Franco L, Alba E, et al. Improvement of breast cancer relapse prediction in high risk intervals using artificial neural networks. Breast Cancer Res Treat 2005;94:265–72. 10.1007/s10549-005-9013-y 16254686

[57] Faraggi D, Simon R. A neural network model for survival data. Stat Med 1995;14:73–82. 10.1002/sim.4780140108 7701159

[58] Katzman J, Shaham U, Cloninger A. Deep survival: a deep COX proportional hazards network, 2016.

