# Supplementary material for: Development of a predictive risk model for all-cause mortality in patients with diabetes in Hong Kong
Source: BMJ Open Diabetes Res Care. 2021 Jun 11;9(1):e001950. doi: 10.1136/bmjdrc-2020-001950 (PMC8201981; doi:10.1136/bmjdrc-2020-001950)

Supplementary Table 1. ICD-9 Codes of the comorbidities.

Comorbidities	ICD Code
Ophthalmological Diabetic Complications	250.5
Renal Diabetic Complications	250.4
Peripheral Vascular Disease	250.7
Ischemic Stroke and Transient Ischemic Attack	433.01, 433.11, 433.21, 433.31, 433.81, 433.91, 434.01, 434.91, 436, 435
Intracranial Hemorrhage	430.00, 431.00, 432.00, 432.10, 432.90, 852.00-852.09, 852.20-852.29, 852.40-852.49, 853.00
Atrial Fibrillation	427.31, 429.4
Heart Failure	428.00-428.99
Ischemic Heart Disease	410.00-411.99, 413.00-414.99
All-Cause Sudden Cardiac Death	410.00-410.99, 427.01, 427.4, 427.5
Osteoporosis	733.00
Dementia	290.00-290.99, 331.00, 294.10-294.29, 331.19
Chronic Obstructive Pulmonary Disease	490.00-496.99
Hypertension	491.00-492.99
Cancer	140.00-209.99

Supplementary Table 2. Formulae for the measures of variability.

Score	Definition
Non-Normalized Score	
Standard deviation	
Absolute successive variability score	$\frac{100 * number\ of\ measurements > 0.5}{number\ of\ measurements}$
Percentage successive variability score	$\frac{100 * number\ of\ measurements > 10\%\ of\ previous\ measurement}{number\ of\ measurements}$
Normalized Score	
Normalized absolute successive variability score	$\frac{100 * number\ of\ measurements > 0.5}{number\ of\ measurements * individual\ mean}$
Normalized percentage successive variability score	$\frac{100 * number\ of\ measurements > 10\%\ of\ previous\ measurement}{number\ of\ measurements * individual\ mean}$
Coefficient of Variation	$\frac{SD}{individual\ mean}$
SD/Initial	$\frac{SD}{individual\ initial\ value}$
Variability Independent of Mean	$\frac{SD}{individual\ mean^{\frac{\ln(population\ SD)}{\ln(population\ mean)}}}$

Supplementary Table 3. Quartile cut-offs and predictive value comparison in fasting blood glucose (FBG) variability measures.

Quartile	Hazard Ratio	95% Confidence Interval	P-Value	AUC
Absolute Successive Variability Score				
Q2	1.11	[1.08, 1.15]	< 0.0001	0.515
Q3	1.24	[1.21, 1.28]	< 0.0001	0.531
Q4	1.49	[1.45, 1.53]	< 0.0001	0.558
Percentage Successive Variability Score				
Q2	1.17	[1.13, 1.21]	< 0.0001	0.520
Q3	1.29	[1.25, 1.33]	< 0.0001	0.534
Q4	1.67	[1.63, 1.72]	< 0.0001	0.574
Standard Deviation				
Q2	1.14	[1.10, 1.17]	< 0.0001	0.517
Q3	1.33	[1.29, 1.37]	< 0.0001	0.539
Q4	1.78	[1.73, 1.83]	< 0.0001	0.583
Normalized Absolute Successive Variability Score				
Q2	1.05	[1.01, 1.08]	0.011	0.506
Q3	1.13	[1.10, 1.17]	< 0.0001	0.518
Q4	1.44	[1.40, 1.47]	< 0.0001	0.553
Normalized Percentage Successive Variability Score				
Q2	1.08	[1.04, 1.11]	< 0.0001	0.509
Q3	1.25	[1.21, 1.29]	< 0.0001	0.530
Q4	1.57	[1.53, 1.61]	< 0.0001	0.565
Standard Deviation/ Initial FBG				
Q2	1.13	[1.09, 1.17]	< 0.0001	0.515
Q3	1.36	[1.31, 1.40]	< 0.0001	0.540
Q4	1.90	[1.86, 1.97]	< 0.0001	0.593
Coefficient of Variation				
Q2	1.12	[1.09, 1.16]	< 0.0001	0.514
Q3	1.35	[1.31, 1.40]	< 0.0001	0.540
Q4	1.96	[1.91, 2.01]	< 0.0001	0.596
Variability Independent of Mean				
Q2	1.12	[1.08, 1.16]	< 0.0001	0.514
Q3	1.35	[1.31, 1.41]	< 0.0001	0.540
Q4	1.92	[1.87, 1.98]	< 0.0001	0.594

Supplementary Table 4. Quartile cut-offs and predictive value comparison in HbA1c variability measures.

	Hazard Ratio	95% Confidence Interval	P-Value	AUC
Absolute Successive Variability Score				
Q2	1.17	[1.13, 1.20]	< 0.0001	0.521
Q3	1.23	[1.19, 1.27]	< 0.0001	0.529
Q4	1.46	[1.42, 1.50]	< 0.0001	0.555
Percentage Successive Variability Score				
Q2	1.17	[1.14, 1.21]	< 0.0001	0.522
Q3	1.31	[1.27, 1.35]	< 0.0001	0.538
Q4	1.50	[1.46, 1.54]	< 0.0001	0.558
Standard Deviation				
Q2	1.07	[1.04, 1.10]	< 0.0001	0.509
Q3	1.20	[1.16, 1.23]	< 0.0001	0.525
Q4	1.58	[1.54, 1.62]	< 0.0001	0.568
Normalized Absolute Successive Variability Score				
Q2	1.16	[1.13, 1.20]	< 0.0001	0.520
Q3	1.25	[1.21, 1.28]	< 0.0001	0.531
Q4	1.45	[1.41, 1.49]	< 0.0001	0.554
Normalized Percentage Successive Variability Score				
Q2	1.20	[1.16, 1.23]	< 0.0001	0.525
Q3	1.29	[1.26, 1.33]	< 0.0001	0.536
Q4	1.51	[1.47, 1.55]	< 0.0001	0.559
Standard Deviation/ Initial HbA1c				
Q2	1.08	[1.05, 1.11]	< 0.0001	0.510
Q3	1.24	[1.20, 1.28]	< 0.0001	0.529
Q4	1.62	[1.57, 1.66]	< 0.0001	0.570
Coefficient of Variation				
Q2	1.06	[1.03, 1.09]	< 0.001	0.507
Q3	1.26	[1.22, 1.30]	< 0.0001	0.532
Q4	1.63	[1.59, 1.68]	< 0.0001	0.572
Variability Independent of Mean				
Q2	1.07	[1.03, 1.10]	< 0.0001	0.508
Q3	1.25	[1.21, 1.28]	< 0.0001	0.530
Q4	1.63	[1.59, 1.67]	< 0.0001	0.572

Supplementary Table 5. Variable importance ranking generated by RSF model

**Characteristics**	**Importance**
Neutrophil-lymphocyte ratio	0.1373
Age	0.0215
Anemia	0.0167
Standard deviation of fasting blood glucose	0.0112
Heart failure	0.0061
Standard deviation of HbA1c	0.0054
Mean fasting blood glucose	0.0038
Hypertension	0.0030
High density lipoprotein-cholesterol	0.0022

Supplementary Figure 1A. Graphical representation of quartile hazard ratios from fasting blood glucose variability measures.

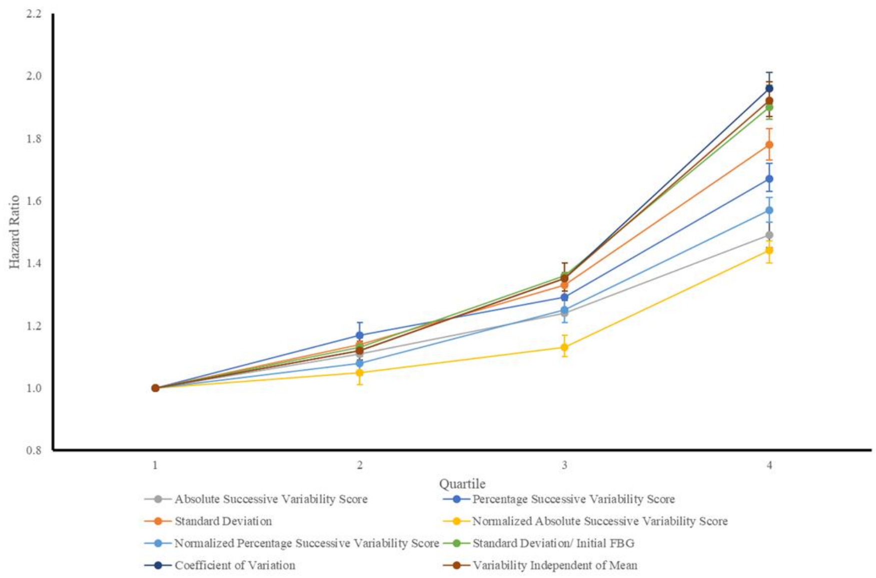

Supplementary Figure 1B. Graphical representation of quartile hazard ratios from HbA1c variability measures.

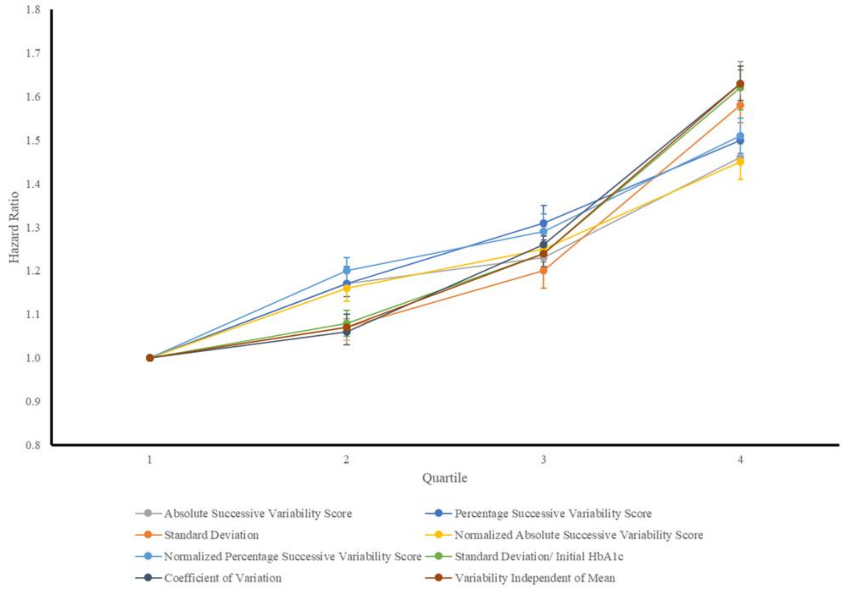

Supplementary Figure 2. Receiver operating characteristic curve and area under the curve (AUC) of the score-based predictive risk model for all-cause mortality.

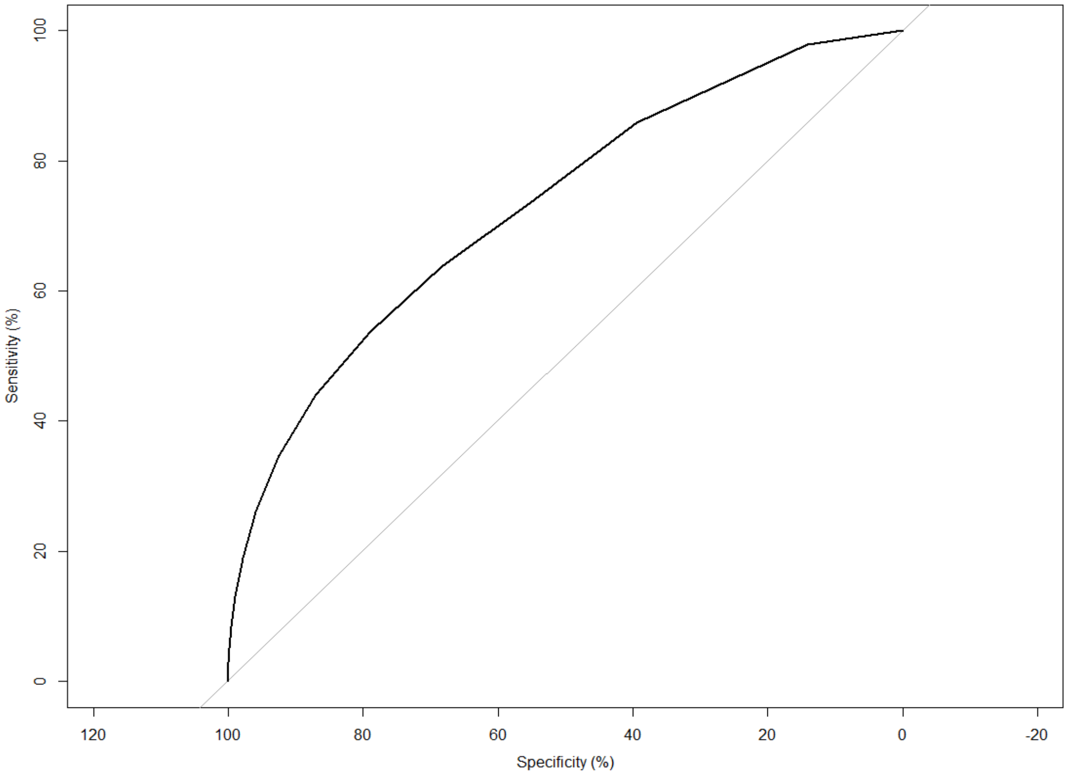

Supplementary Figure 3. Kaplan–Meier curve of all-cause mortality.

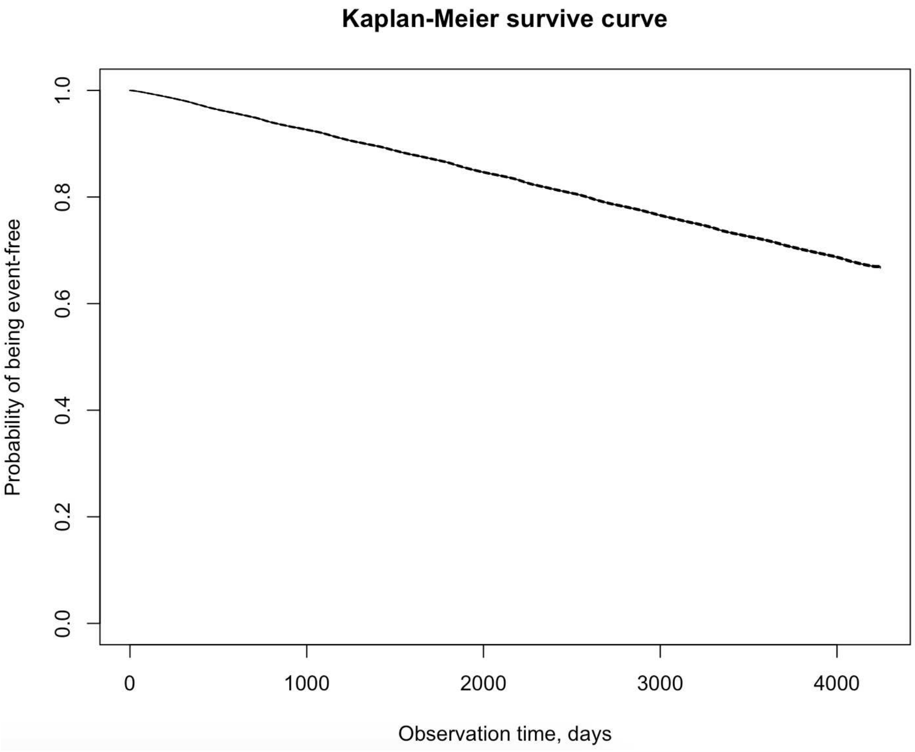

Supplementary Figure 4. Survival curve generated by multivariate Cox regression.

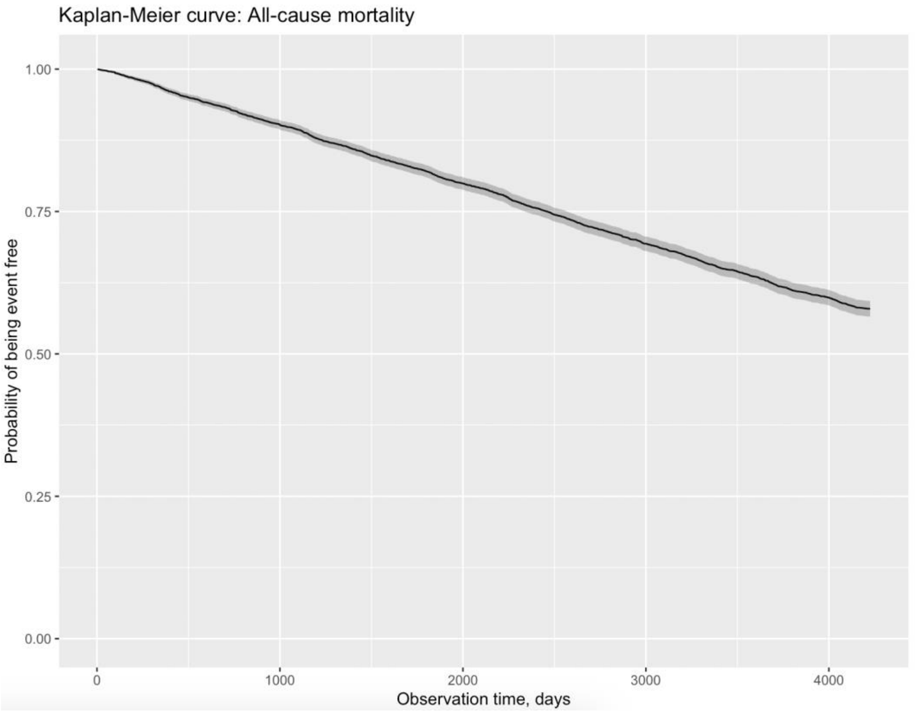

Supplementary Figure 5. Selecting optimal tree number for RSF model.

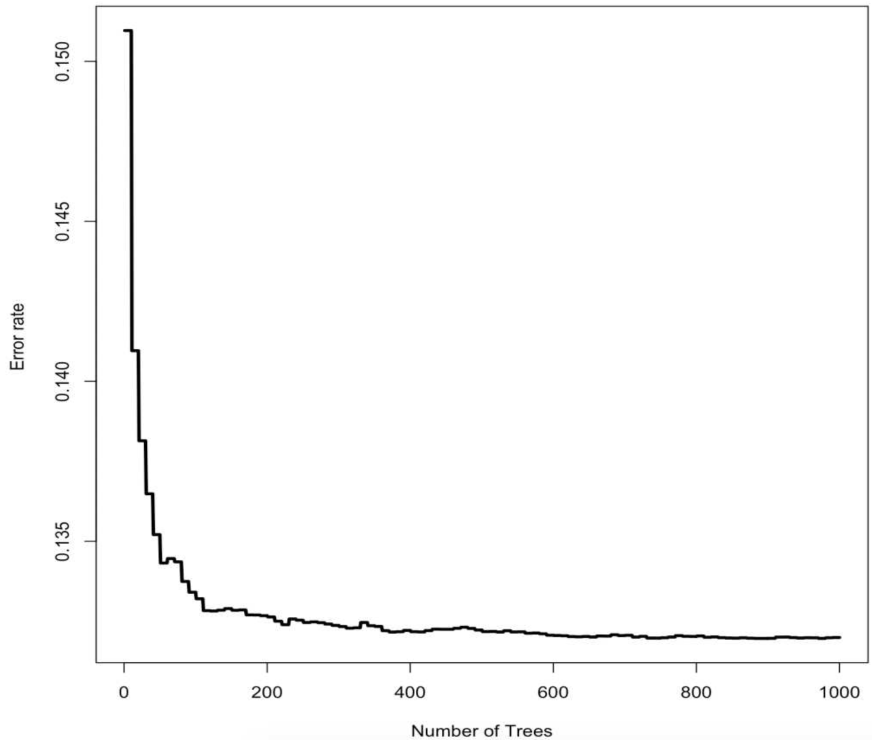

Supplementary Figure 6. Predicted OOB survivals and the cumulative hazard with RSF model.

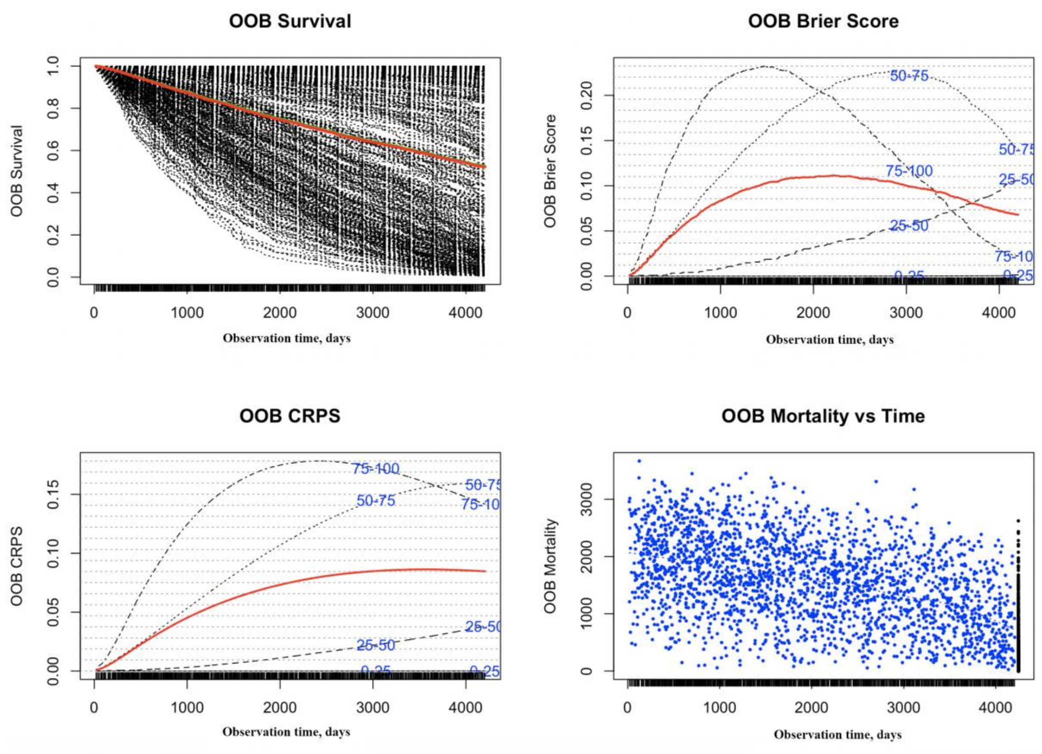

Supplementary Figure 7. Predicted OOB survivals and the cumulative hazard with RSF model.

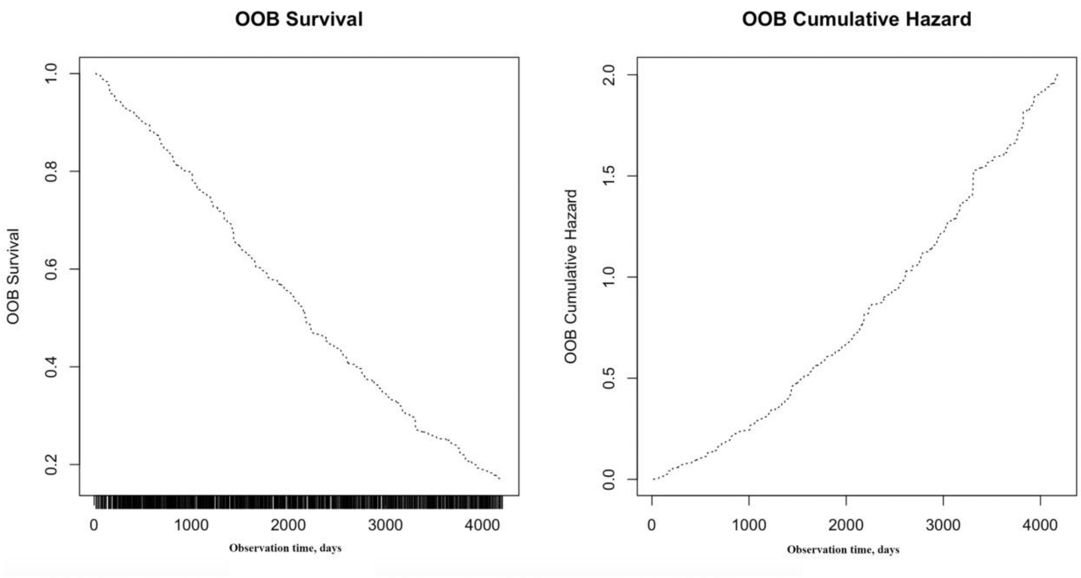

Supplementary Figure 8. RSF model's reproducibility by comparing one-fold 'test' predicted survivals.

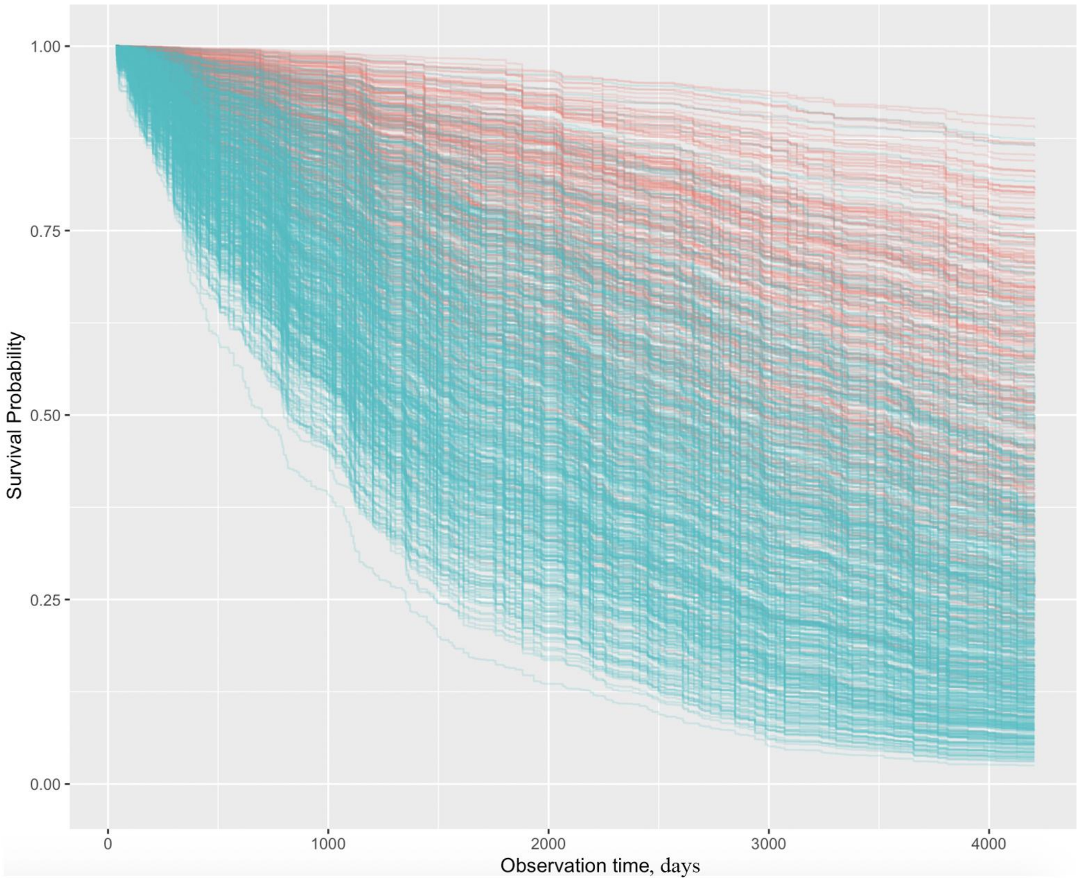

Supplement: Supplementary data [file bmjdrc-2020-001950supp001.pdf]
